# Supplementary figures and images for: Improving the COI DNA barcoding library for Neotropical phlebotomine sand flies (Diptera: Psychodidae)
Source: Parasit Vectors. 2023 Jun 12;16:198. doi: 10.1186/s13071-023-05807-z (PMC10259023; doi:10.1186/s13071-023-05807-z)

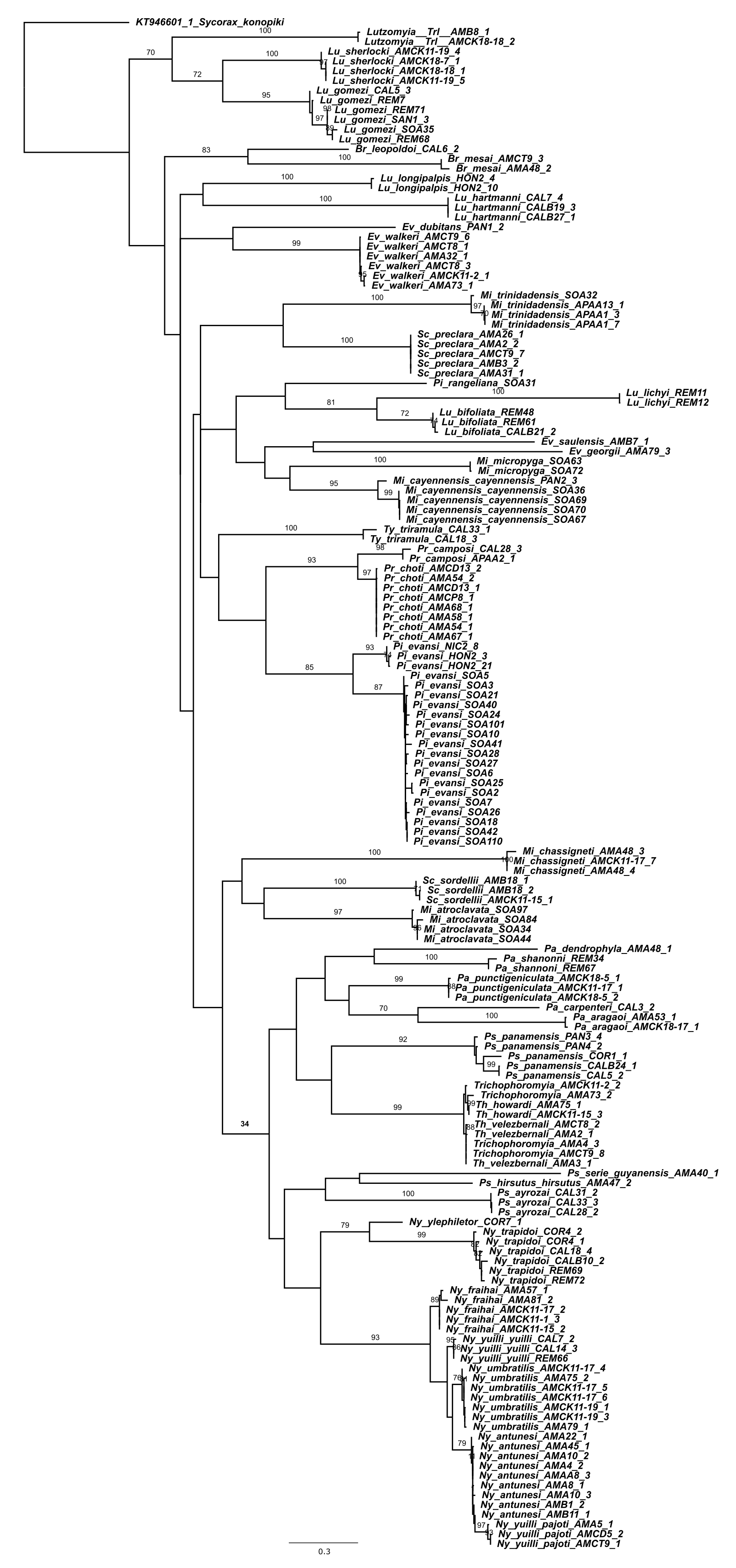

Supplement: Supplementary file 1 — Additional file 1: Figure S1. Phylogenetic gene tree based on COI DNA barcode sequences of Neotropical sand flies. Numbers near nodes indicate bootstrap values above 70, except the clade comprising Psychodopygina species, which is highlighted in bold. [file 13071_2023_5807_MOESM1_ESM.png]
