# Supplementary material for: Improving the COI DNA barcoding library for Neotropical phlebotomine sand flies (Diptera: Psychodidae)
Source: Parasit Vectors. 2023 Jun 12;16:198. doi: 10.1186/s13071-023-05807-z (PMC10259023; doi:10.1186/s13071-023-05807-z)
Supplement: Supplementary file 2 — Additional file 2: Table S1. Sample IDs, BOLD processes IDs, GenBank accession numbers, nominal species, sex of the specimens, and collection sites of sand fly species from the Neotropical region analyzed in this study. [file 13071_2023_5807_MOESM2_ESM.docx]

**Table S1.** Sample IDs, BOLD processes IDs, GenBank accession numbers, nominal species, sex of the specimens, and collection sites of sand fly species from the Neotropical region analysed in this study.

| **Sample ID** | **BOLD processes IDs** | **GenBank accessions** | **Species** | **Sex** | ***Country*** | **Department** | **Municipality** |
| --- | --- | --- | --- | --- | --- | --- | --- |
| AMCT9.8 | CLBAR001-22 | OP964352 | *Trichophoromyia* sp. | F | Colombia | Amazonas | Puerto Nariño |
| AMCT8.2 | CLBAR002-22 | OP964360 | *Trichophoromyia velezbernali* | M | Colombia | Amazonas | Puerto Nariño |
| AMCK11-2.2 | CLBAR003-22 | OP964355 | *Trichophoromyia* sp. | F | Colombia | Amazonas | Leticia |
| AMA73.2 | CLBAR004-22 | OP964354 | *Trichophoromyia* sp. | F | Colombia | Amazonas | Leticia |
| AMA4.3 | CLBAR005-22 | OP964353 | *Trichophoromyia* sp. | F | Colombia | Amazonas | Puerto Nariño |
| AMA3.1 | CLBAR006-22 | OP964358 | *Trichophoromyia velezbernali* | M | Colombia | Amazonas | Puerto Nariño |
| AMA2.1 | CLBAR007-22 | OP964359 | *Trichophoromyia velezbernali* | M | Colombia | Amazonas | Puerto Nariño |
| AMCK11-15.3 | CLBAR008-22 | OP964357 | *Trichophoromyia howardi* | M | Colombia | Amazonas | Leticia |
| AMA75.1 | CLBAR009-22 | OP964356 | *Trichophoromyia howardi* | M | Colombia | Amazonas | Leticia |
| AMCK11-15.1 | CLBAR010-22 | OP964350 | *Sciopemyia sordellii* | M | Colombia | Amazonas | Leticia |
| AMB18.2 | CLBAR011-22 | OP964351 | *Sciopemyia sordellii* | F | Colombia | Amazonas | Leticia |
| AMB18.1 | CLBAR012-22 | OP964349 | *Sciopemyia sordellii* | M | Colombia | Amazonas | Leticia |
| AMCT9.7 | CLBAR013-22 | OP964345 | *Sciopemyia preclara* | F | Colombia | Amazonas | Puerto Nariño |
| AMB3.2 | CLBAR014-22 | OP964348 | *Sciopemyia preclara* | F | Colombia | Amazonas | Puerto Nariño |
| AMA31.1 | CLBAR015-22 | OP964344 | *Sciopemyia preclara* | M | Colombia | Amazonas | Puerto Nariño |
| AMA26.1 | CLBAR016-22 | OP964347 | *Sciopemyia preclara* | F | Colombia | Amazonas | Puerto Nariño |
| AMA2.2 | CLBAR017-22 | OP964346 | *Sciopemyia preclara* | M | Colombia | Amazonas | Puerto Nariño |
| AMA40.1 | CLBAR018-22 | OP964334 | *Psychodopygus* Guyanensis Series | F | Colombia | Amazonas | Puerto Nariño |
| PAN4.2 | CLBAR020-22 | OP964339 | *Psychodopygus panamensis* | F | Panama | Panama Oeste | Capira-Ollas Arriba |
| PAN3.4 | CLBAR021-22 | OP964340 | *Psychodopygus panamensis* | F | Panama | Panama Oeste | Capira-Ollas Arriba |
| COR1.1 | CLBAR022-22 | OP964343 | *Psychodopygus panamensis* | F | Costa Rica | Limón | San Vicente |
| CALB24.1 | CLBAR023-22 | OP964342 | *Psychodopygus panamensis* | M | Colombia | Caldas | Victoria |
| CAL5.2 | CLBAR024-22 | OP964341 | *Psychodopygus panamensis* | F | Colombia | Caldas | Samaná |
| AMA47.2 | CLBAR025-22 | OP964338 | *Psychodopygus hirsutus hirsutus* | F | Colombia | Amazonas | Puerto Nariño |
| CAL33.3 | CLBAR026-22 | OP964337 | *Psychodopygus ayrozai* | M | Colombia | Caldas | Samaná |
| CAL31.2 | CLBAR027-22 | OP964336 | *Psychodopygus ayrozai* | F | Colombia | Caldas | Samaná |
| CAL28.2 | CLBAR028-22 | OP964335 | *Psychodopygus ayrozai* | F | Colombia | Caldas | Samaná |
| AMCP8.1 | CLBAR030-22 | OP964324 | *Pressatia choti* | F | Colombia | Amazonas | Puerto Nariño |
| AMCD13.2 | CLBAR031-22 | OP964323 | *Pressatia choti* | M | Colombia | Amazonas | Puerto Nariño |
| AMCD13.1 | CLBAR032-22 | OP964317 | *Pressatia choti* | M | Colombia | Amazonas | Puerto Nariño |
| AMA68.1 | CLBAR033-22 | OP964318 | *Pressatia choti* | M | Colombia | Amazonas | Puerto Nariño |
| AMA67.1 | CLBAR034-22 | OP964319 | *Pressatia choti* | F | Colombia | Amazonas | Puerto Nariño |
| AMA58.1 | CLBAR035-22 | OP964320 | *Pressatia choti* | F | Colombia | Amazonas | Puerto Nariño |
| AMA54.2 | CLBAR036-22 | OP964321 | *Pressatia choti* | F | Colombia | Amazonas | Puerto Nariño |
| AMA54.1 | CLBAR037-22 | OP964322 | *Pressatia choti* | M | Colombia | Amazonas | Puerto Nariño |
| CAL28.3 | CLBAR038-22 | OP964316 | *Pressatia camposi* | M | Colombia | Caldas | Samaná |
| APAA2.1 | CLBAR039-22 | OP964315 | *Pressatia camposi* | F | Colombia | Antioquia | Apartado |
| SOA31 | CLBAR040-22 | OP964314 | *Pintomyia rangeliana* | F | Colombia | Sucre | Ovejas |
| SOA7 | CLBAR041-22 | OP964293 | *Pintomyia evansi* | M | Colombia | Sucre | Ovejas |
| SOA6 | CLBAR042-22 | OP964304 | *Pintomyia evansi* | F | Colombia | Sucre | Ovejas |
| SOA5 | CLBAR043-22 | OP964303 | *Pintomyia evansi* | M | Colombia | Sucre | Ovejas |
| SOA42 | CLBAR044-22 | OP964302 | *Pintomyia evansi* | F | Colombia | Sucre | Ovejas |
| SOA41 | CLBAR045-22 | OP964301 | *Pintomyia evansi* | F | Colombia | Sucre | Ovejas |
| SOA40 | CLBAR046-22 | OP964300 | *Pintomyia evansi* | F | Colombia | Sucre | Ovejas |
| SOA3 | CLBAR047-22 | OP964299 | *Pintomyia evansi* | F | Colombia | Sucre | Ovejas |
| SOA28 | CLBAR048-22 | OP964298 | *Pintomyia evansi* | F | Colombia | Sucre | Ovejas |
| SOA27 | CLBAR049-22 | OP964297 | *Pintomyia evansi* | M | Colombia | Sucre | Ovejas |
| SOA26 | CLBAR050-22 | OP964296 | *Pintomyia evansi* | M | Colombia | Sucre | Ovejas |
| SOA25 | CLBAR051-22 | OP964295 | *Pintomyia evansi* | M | Colombia | Sucre | Ovejas |
| SOA24 | CLBAR052-22 | OP964294 | *Pintomyia evansi* | F | Colombia | Sucre | Ovejas |
| SOA21 | CLBAR053-22 | OP964313 | *Pintomyia evansi* | M | Colombia | Sucre | Ovejas |
| SOA2 | CLBAR054-22 | OP964312 | *Pintomyia evansi* | F | Colombia | Sucre | Ovejas |
| SOA18 | CLBAR055-22 | OP964311 | *Pintomyia evansi* | F | Colombia | Sucre | Ovejas |
| SOA110 | CLBAR056-22 | OP964310 | *Pintomyia evansi* | M | Colombia | Sucre | Ovejas |
| SOA101 | CLBAR057-22 | OP964309 | *Pintomyia evansi* | M | Colombia | Sucre | Ovejas |
| SOA10 | CLBAR058-22 | OP964308 | *Pintomyia evansi* | M | Colombia | Sucre | Ovejas |
| NIC2.8 | CLBAR059-22 | OP964307 | *Pintomyia evansi* | F | Nicaragua | León | Rota |
| HON2.3 | CLBAR060-22 | OP964306 | *Pintomyia evansi* | F | Honduras | Valle | Amapola - El Caracol |
| HON2.21 | CLBAR061-22 | OP964305 | *Pintomyia evansi* | F | Honduras | Valle | Amapola - El Caracol |
| REM34 | CLBAR062-22 | OP964332 | *Psathyromyia shannoni* | F | Colombia | Antioquia | Remedios |
| REM67 | CLBAR063-22 | OP964333 | *Psathyromyia shannoni* | F | Colombia | Antioquia | Remedios |
| AMCK18-5.2 | CLBAR064-22 | OP964331 | *Psathyromyia punctigeniculata* | M | Colombia | Amazonas | Leticia |
| AMCK18-5.1 | CLBAR065-22 | OP964330 | *Psathyromyia punctigeniculata* | M | Colombia | Amazonas | Leticia |
| AMCK11-17.1 | CLBAR066-22 | OP964329 | *Psathyromyia punctigeniculata* | M | Colombia | Amazonas | Leticia |
| AMA48.1 | CLBAR067-22 | OP964328 | *Psathyromyia dendrophyla* | M | Colombia | Amazonas | Puerto Nariño |
| CAL3.2 | CLBAR068-22 | OP964327 | *Psathyromyia carpenteri* | F | Colombia | Caldas | Norcasia |
| AMCK18-17.1 | CLBAR069-22 | OP964326 | *Psathyromyia aragaoi* | M | Colombia | Amazonas | Leticia |
| AMA53.1 | CLBAR070-22 | OP964325 | *Psathyromyia aragaoi* | F | Colombia | Amazonas | Puerto Nariño |
| REM66 | CLBAR071-22 | OP964291 | *Nyssomyia yuilli yuilli* | F | Colombia | Antioquia | Remedios |
| CAL7.2 | CLBAR072-22 | OP964292 | *Nyssomyia yuilli yuilli* | M | Colombia | Caldas | Samaná |
| CAL14.3 | CLBAR073-22 | OP964290 | *Nyssomyia yuilli yuilli* | F | Colombia | Caldas | Norcasia |
| AMCT9.1 | CLBAR074-22 | OP964289 | *Nyssomyia yuilli pajoti* | F | Colombia | Amazonas | Puerto Nariño |
| AMCD5.2 | CLBAR075-22 | OP964288 | *Nyssomyia yuilli pajoti* | F | Colombia | Amazonas | Puerto Nariño |
| AMA5.1 | CLBAR076-22 | OP964287 | *Nyssomyia yuilli pajoti* | F | Colombia | Amazonas | Puerto Nariño |
| COR7.1 | CLBAR077-22 | OP964286 | *Nyssomyia ylephiletor* | F | Costa Rica | Limón | Sibuju |
| AMCK11-19.3 | CLBAR078-22 | OP964285 | *Nyssomyia umbratilis* | M | Colombia | Amazonas | Leticia |
| AMCK11-19.1 | CLBAR079-22 | OP964284 | *Nyssomyia umbratilis* | M | Colombia | Amazonas | Leticia |
| AMCK11-17.6 | CLBAR080-22 | OP964279 | *Nyssomyia umbratilis* | F | Colombia | Amazonas | Leticia |
| AMCK11-17.5 | CLBAR081-22 | OP964283 | *Nyssomyia umbratilis* | M | Colombia | Amazonas | Leticia |
| AMCK11-17.4 | CLBAR082-22 | OP964282 | *Nyssomyia umbratilis* | M | Colombia | Amazonas | Leticia |
| AMA79.1 | CLBAR083-22 | OP964281 | *Nyssomyia umbratilis* | M | Colombia | Amazonas | Leticia |
| AMA75.2 | CLBAR084-22 | OP964280 | *Nyssomyia umbratilis* | M | Colombia | Amazonas | Leticia |
| REM72 | CLBAR085-22 | OP964276 | *Nyssomyia trapidoi* | F | Colombia | Antioquia | Remedios |
| REM69 | CLBAR086-22 | OP964277 | *Nyssomyia trapidoi* | F | Colombia | Antioquia | Remedios |
| COR4.2 | CLBAR087-22 | OP964274 | *Nyssomyia trapidoi* | F | Costa Rica | Limón | Sibuju |
| COR4.1 | CLBAR088-22 | OP964278 | *Nyssomyia trapidoi* | F | Costa Rica | Limón | Sibuju |
| CALB10.2 | CLBAR089-22 | OP964273 | *Nyssomyia trapidoi* | F | Colombia | Caldas | Samaná |
| CAL18.4 | CLBAR090-22 | OP964275 | *Nyssomyia trapidoi* | F | Colombia | Caldas | Samaná |
| AMCK11-17.2 | CLBAR091-22 | OP964269 | *Nyssomyia fraihai* | F | Colombia | Amazonas | Leticia |
| AMCK11-15.2 | CLBAR092-22 | OP964272 | *Nyssomyia fraihai* | F | Colombia | Amazonas | Leticia |
| AMCK11-1.3 | CLBAR093-22 | OP964268 | *Nyssomyia fraihai* | F | Colombia | Amazonas | Leticia |
| AMA81.2 | CLBAR094-22 | OP964271 | *Nyssomyia fraihai* | F | Colombia | Amazonas | Leticia |
| AMA57.1 | CLBAR095-22 | OP964270 | *Nyssomyia fraihai* | F | Colombia | Amazonas | Puerto Nariño |
| AMB11.1 | CLBAR096-22 | OP964260 | *Nyssomyia antunesi* | M | Colombia | Amazonas | Puerto Nariño |
| AMB1.2 | CLBAR097-22 | OP964267 | *Nyssomyia antunesi* | F | Colombia | Amazonas | Puerto Nariño |
| AMAA8.3 | CLBAR098-22 | OP964259 | *Nyssomyia antunesi* | F | Colombia | Amazonas | Puerto Nariño |
| AMA8.1 | CLBAR099-22 | OP964266 | *Nyssomyia antunesi* | M | Colombia | Amazonas | Puerto Nariño |
| AMA45.1 | CLBAR100-22 | OP964265 | *Nyssomyia antunesi* | M | Colombia | Amazonas | Puerto Nariño |
| AMA4.2 | CLBAR101-22 | OP964264 | *Nyssomyia antunesi* | M | Colombia | Amazonas | Puerto Nariño |
| AMA22.1 | CLBAR102-22 | OP964263 | *Nyssomyia antunesi* | F | Colombia | Amazonas | Puerto Nariño |
| AMA10.3 | CLBAR103-22 | OP964262 | *Nyssomyia antunesi* | F | Colombia | Amazonas | Puerto Nariño |
| AMA10.2 | CLBAR104-22 | OP964261 | *Nyssomyia antunesi* | F | Colombia | Amazonas | Puerto Nariño |
| SOA32 | CLBAR105-22 | OP964256 | *Micropygomyia trinidadensis* | F | Colombia | Sucre | Ovejas |
| APAA13.1 | CLBAR106-22 | OP964258 | *Micropygomyia trinidadensis* | F | Colombia | Antioquia | Apartadó |
| APAA1.7 | CLBAR107-22 | OP964255 | *Micropygomyia trinidadensis* | M | Colombia | Antioquia | Apartadó |
| APAA1.3 | CLBAR108-22 | OP964257 | *Micropygomyia trinidadensis* | F | Colombia | Antioquia | Apartadó |
| SOA72 | CLBAR109-22 | OP964253 | *Micropygomyia micropyga* | F | Colombia | Sucre | Ovejas |
| SOA63 | CLBAR110-22 | OP964254 | *Micropygomyia micropyga* | M | Colombia | Sucre | Ovejas |
| AMCK11-17.7 | CLBAR111-22 | OP964250 | *Micropygomyia chassigneti* | F | Colombia | Amazonas | Leticia |
| AMA48.4 | CLBAR112-22 | OP964252 | *Micropygomyia chassigneti* | F | Colombia | Amazonas | Puerto Nariño |
| AMA48.3 | CLBAR113-22 | OP964251 | *Micropygomyia chassigneti* | M | Colombia | Amazonas | Puerto Nariño |
| SOA70 | CLBAR114-22 | OP964246 | *Micropygomyia cayennensis cayennensis* | F | Colombia | Sucre | Ovejas |
| SOA69 | CLBAR115-22 | OP964245 | *Micropygomyia cayennensis cayennensis* | M | Colombia | Sucre | Ovejas |
| SOA67 | CLBAR116-22 | OP964249 | *Micropygomyia cayennensis cayennensis* | M | Colombia | Sucre | Ovejas |
| SOA36 | CLBAR117-22 | OP964248 | *Micropygomyia cayennensis cayennensis* | F | Colombia | Sucre | Ovejas |
| PAN2.3 | CLBAR118-22 | OP964247 | *Micropygomyia cayennensis cayennensis* | F | Panama | Panama Oeste | Capira-Ollas Arriba |
| SOA97 | CLBAR119-22 | OP964244 | *Micropygomyia atroclavata* | F | Colombia | Sucre | Ovejas |
| SOA84 | CLBAR120-22 | OP964243 | *Micropygomyia atroclavata* | F | Colombia | Sucre | Ovejas |
| SOA44 | CLBAR121-22 | OP964242 | *Micropygomyia atroclavata* | M | Colombia | Sucre | Ovejas |
| SOA34 | CLBAR122-22 | OP964241 | *Micropygomyia atroclavata* | F | Colombia | Sucre | Ovejas |
| AMCK18-18.2 | CLBAR123-22 | OP964239 | *Lutzomyia* (*Tricholateralis*) sp. | F | Colombia | Amazonas | Leticia |
| AMB8.1 | CLBAR124-22 | OP964240 | *Lutzomyia* (*Tricholateralis*) sp. | F | Colombia | Amazonas | Puerto Nariño |
| AMCK18-7.1 | CLBAR125-22 | OP964235 | *Lutzomyia sherlocki* | M | Colombia | Amazonas | Leticia |
| AMCK18-18.1 | CLBAR126-22 | OP964237 | *Lutzomyia sherlocki* | M | Colombia | Amazonas | Leticia |
| AMCK11-19.5 | CLBAR127-22 | OP964236 | *Lutzomyia sherlocki* | F | Colombia | Amazonas | Leticia |
| AMCK11-19.4 | CLBAR128-22 | OP964238 | *Lutzomyia sherlocki* | F | Colombia | Amazonas | Leticia |
| HON2.4 | CLBAR129-22 | OP964234 | *Lutzomyia longipalpis* | M | Honduras | Valle | Amapola - El Caracol |
| HON2.10 | CLBAR130-22 | OP964233 | *Lutzomyia longipalpis* | M | Honduras | Valle | Amapola - El Caracol |
| REM12 | CLBAR131-22 | OP964231 | *Lutzomyia lichyi* | F | Colombia | Antioquia | Remedios |
| REM11 | CLBAR132-22 | OP964232 | *Lutzomyia lichyi* | F | Colombia | Antioquia | Remedios |
| CALB27.1 | CLBAR133-22 | OP964229 | *Lutzomyia hartmanni* | M | Colombia | Caldas | Victoria |
| CALB19.3 | CLBAR134-22 | OP964230 | *Lutzomyia hartmanni* | F | Colombia | Caldas | Victoria |
| CAL7.4 | CLBAR135-22 | OP964228 | *Lutzomyia hartmanni* | F | Colombia | Caldas | Samaná |
| SOA35 | CLBAR136-22 | OP964223 | *Lutzomyia gomezi* | F | Colombia | Sucre | Ovejas |
| SAN1.3 | CLBAR137-22 | OP964227 | *Lutzomyia gomezi* | F | Colombia | Magdalena | Santa Marta |
| REM71 | CLBAR138-22 | OP964222 | *Lutzomyia gomezi* | F | Colombia | Antioquia | Remedios |
| REM7 | CLBAR139-22 | OP964226 | *Lutzomyia gomezi* | F | Colombia | Antioquia | Remedios |
| REM68 | CLBAR140-22 | OP964225 | *Lutzomyia gomezi* | F | Colombia | Antioquia | Remedios |
| CAL5.3 | CLBAR141-22 | OP964224 | *Lutzomyia gomezi* | F | Colombia | Caldas | Samaná |
| REM61 | CLBAR142-22 | OP964220 | *Lutzomyia bifoliata* | F | Colombia | Antioquia | Remedios |
| REM48 | CLBAR143-22 | OP964221 | *Lutzomyia bifoliata* | F | Colombia | Antioquia | Remedios |
| CALB21.2 | CLBAR144-22 | OP964219 | *Lutzomyia bifoliata* | F | Colombia | Caldas | Victoria |
| AMCT9.6 | CLBAR145-22 | OP964214 | *Evandromyia walkeri* | M | Colombia | Amazonas | Puerto Nariño |
| AMCT8.3 | CLBAR146-22 | OP964218 | *Evandromyia walkeri* | M | Colombia | Amazonas | Puerto Nariño |
| AMCT8.1 | CLBAR147-22 | OP964213 | *Evandromyia walkeri* | F | Colombia | Amazonas | Puerto Nariño |
| AMCK11-2.1 | CLBAR148-22 | OP964217 | *Evandromyia walkeri* | F | Colombia | Amazonas | Leticia |
| AMA73.1 | CLBAR149-22 | OP964216 | *Evandromyia walkeri* | M | Colombia | Amazonas | Leticia |
| AMA32.1 | CLBAR150-22 | OP964215 | *Evandromyia walkeri* | F | Colombia | Amazonas | Puerto Nariño |
| AMB7.1 | CLBAR151-22 | OP964212 | *Evandromyia saulensis* | F | Colombia | Amazonas | Puerto Nariño |
| AMA79.3 | CLBAR152-22 | OP964211 | *Evandromyia georgii* | F | Colombia | Amazonas | Leticia |
| PAN1.2 | CLBAR153-22 | OP964210 | *Evandromyia dubitans* | F | Panama | Panama Oeste | Capira-Ollas Arriba |
| AMCT9.3 | CLBAR154-22 | OP964209 | *Brumptomyia mesai* | M | Colombia | Amazonas | Puerto Nariño |
| AMA48.2 | CLBAR155-22 | OP964208 | *Brumptomyia mesai* | M | Colombia | Amazonas | Puerto Nariño |
| CAL6.2 | CLBAR156-22 | OP964207 | *Brumptomyia leopoldoi* | M | Colombia | Caldas | Norcasia |
| CAL18.3 | CLBAR157-22 | OP964362 | *Trichopygomyia triramula* | M | Colombia | Caldas | Samaná |
| CAL33.1 | CLBAR158-22 | OP964361 | *Trichopygomyia triramula* | F | Colombia | Caldas | Samaná |
